# Use of an UHPLC-MS/MS Method for Determination of Kuraridin and Characterization of Its Metabolites in Rat Plasma after Oral Administration

**DOI:** 10.3390/molecules23020132

**Published:** 2018-01-24

**Authors:** Yi Liu, Lei Chen, Wei Cai, Lin-lin Zhao, Zhi-xian Mo

**Affiliations:** 1 School of Traditional Chinese Medicine, Southern Medical University, Guangzhou 510515, China; liuyi099@163.com; 2 Key Laboratory of Digital Quality Evaluation of Chinese Materia Medica of State Administration of TCM, China; Engineering & Technology Research Center for Chinese Materia Medical Quality of the Universities of Guangdong Province, Guangdong Pharmaceutical University, Guangzhou 510006, China; chenlei0080@163.com (L.C.); zll449158@163.com (L.-l.Z.); 3 Department of Pharmacy, Hunan University of Medicine, Huaihua 418000, China; wei.cai@yale.edu

**Keywords:** kuraridin, UHPLC-MS/MS, rat plasma, metabolites, pharmacokinetic

## Abstract

Kuraridin is an active natural prenylated flavonoid ingredient originating from the well-known traditional Chinese medicine *Sophora flavescens* Ait., that possesses various bioactivities, such as antitumor activity, PLC_γ_1 inhibitory activity, glycosidase inhibitory activity, etc. However, there is no report on the plasma metabolic profile and pharmacokinetic study of kuraridin. The current study was designed to use an ultra-performance liquid chromatography/tandem mass spectrometry (UHPLC-MS/MS) method for the quantification and characterization metabolites in rat plasma after oral administration of kuraridin. A liquid-liquid extraction method with ethyl acetate-acetonitrile (1:3) was used to extract the kuraridin from rat plasma samples. The chromatographic separation was carried out on a Hypersil GOLD UHPLC C18 column equipped with a C18 guard cartridge using a gradient elution with organic solvent-water as mobile phase. Based on comparing the retention times with reference standards or on the basis of MS_2_ fragmentation behaviors, a total of 19 metabolites were identified or tentatively characterized from rat plasma. Under the optimized conditions, the method showed good linearity (r^2^ > 0.99) over the ranges of 1–500 ng/mL for kuraridin. The inter- and intra-day precisions were less than 8.95%, and the accuracy was in the range of −6.27–6.48%. The recovery of kuraridin ranged from 90.1% to 100.4%. The developed UHPLC-MS/MS method was thus successfully applied in the qualitative of metabolites and quantitative analysis of kuraridin in rat plasma.

## 1. Introduction

For thousands of years, the dried roots of *Sophora flavescens* Ait. (*S. flavescens*), also named ‘Kushen’, have been traditionally used in East Asian countries as an herbal medicine for the treatment of eczema, fever, hepatitis, gastrointestinal hemorrhage and skin diseases [1]. Previous study have revealed that prenylated flavonoids from *S. flavescens* exhibit a variety of bioactivities. Kuraridin, one of the major prenylated flavonoids from *S. flavescens*, has a long history of use in China for the treatment of clinical diseases in the form of medicinal fractions or in *S. flavescens* compound preparations [2].

Kuraridin displays a variety of biological activities, such as antitumor activity [3,4], PLC_γ_1 inhibitory activity [5], glycosidase inhibitory activity [6], SGLT inhibitory activity [7], tyrosinase inhibitory activity [8], antibacterial actions [9] and anti-reovirus activity [10], but as far as we know, there is no report on the plasma metabolic profile and pharmacokinetic study of kuraridin. Systematic investigation of drug metabolism and clearance is a critical part of drug development. The knowledge of metabolites formed and pharmacokinetics are very important for evaluating drug therapeutic effects and their rational use in clinical situations. 

Therefore, the current study was designed to use an ultra-performance liquid chromatography/tandem mass spectrometry (UHPLC-MS/MS) method for the quantification of kuraridin and characterization of its metabolites in rat plasma after oral administration. As far as we know, the method was applied in the qualitative of metabolites and quantitative analysis of kuraridin in rat plasma for the first time. And the results would provide a reference for the clinical development and clinical research of kuraridin and *S. flavescens*.

## 2. Results and Discussion

### 2.1. Method Development

The ESI sources in both positive and negative ionization were tested for getting a better response for kuraridin and IS, the result indicated that kuraridin and IS had stronger signal intensity in negative ionization mode.

### 2.2. Pharmacokinetic Method Validation

**Figure 1:**
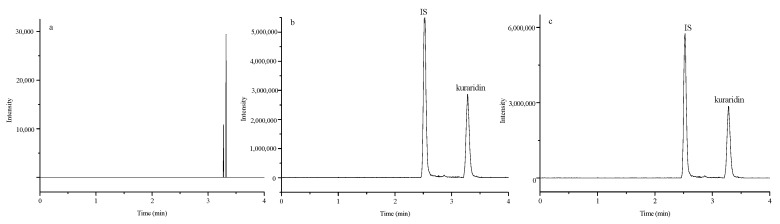
Representative chromatograms of a blank plasma sample (**a**); a blank plasma sample spiked with 15 ng/mL kuraridin and 300 ng/mL IS (**b**); a plasma sample at 15 min after oral administration of kuraridin (**c**) The regression equations, linear ranges, and correlation coefficients for kuraridin were shown in Table 1. The results showed good linearity with correlation coefficients of 0.9931 for kuraridin. The LLOQ for kuraridin were 1 ng/mL with accuracy and precision within 9%.

The typical chromatograms of blank plasma samples, blank plasma samples spiked with kuraridin and IS, plasma samples obtained after an oral dose were shown in Figure 1. Clearly, there were no interfering peak in blank plasma at the retention time of kuraridin and the IS.

The results of the intra- and inter-day precision and accuracy are presented in Table 2. The precision and accuracy for kuraridin at three QC levels were within the acceptable value (±15%), indicating that the method was accurate and reliable for the quantitation of kuraridin in rat plasma.

As shown in Table 3, the extraction recoveries of kuraridin were all more than 90.1% at three level QC samples, and for the IS, it was 91.2 ± 2.9%. The matrix effect of kuraridin at three QC levels were in the range from 84.8% to 87.1%, and for the IS, it was 90.1 ± 3.2%. The result demonstrated that the established method achieved reproducible recoveries and negligible matrix effects.

The result of stability of kuraridin at three QC levels was summarized in Table 4, and suggested that kuraridin were stable under four different storage conditions.

### 2.3. Pharmacokinetic Study

**Figure 2:**
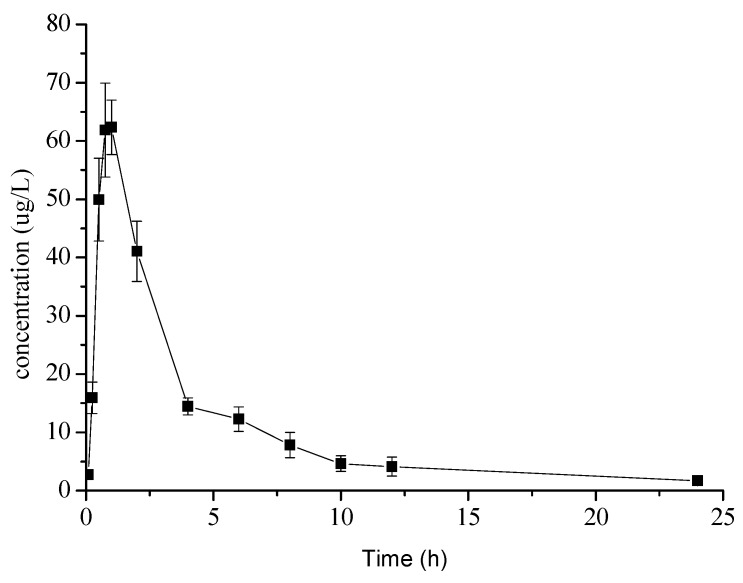
Mean plasma concentration-time profiles of kuraridin after oral administration.

The validated method was used to determine kuraridin in rat plasma after oral administration. The mean plasma concentration-time profiles of kuraridin are presented in Figure 2. The DAS 2.0 software with a non-compartmental approach was used for evaluating the other pharmacokinetic parameters and the main parameters of kuraridin were summarized in Table 5. After oral administration of kuraridin, the plasma concentrations of kuraridin reached maximum plasma concentration (C_max_) at 0.87 h. The t_1/2_ of kuraridin were found to be 5.54 h, respectively. 

### 2.4. Fragmentation Pathway of Kuraridin in Negative Ion Mode

**Scheme 1:**
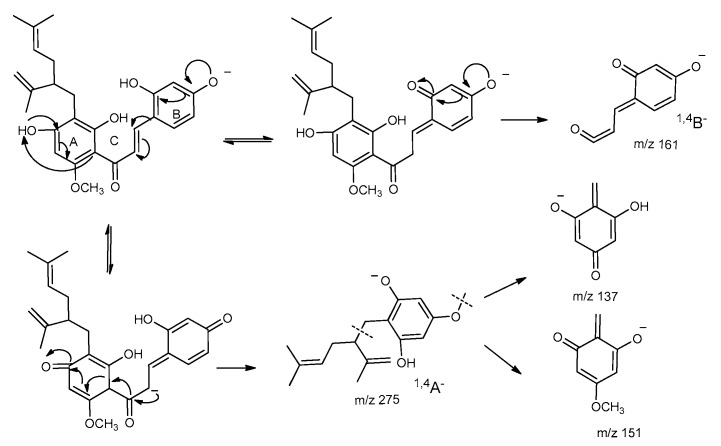
The proposed fragmentation pattern for the [M − H]^−^ ions of kuraridin.

The MS^2^ fragmentation pattern of kuraridin was detected, and it exhibited a [M − H]^–^ ion at *m*/*z* 437.1981 (2.72 ppm, C_26_H_29_O_6_). The fragment ions are labeled in Scheme 1. The parent ion generated ^1,4^A^−^ and ^1,4^B^−^ ions at *m*/*z* 275.1657 (1.54 ppm, C_17_H_23_O_3_) and 161.0236 (0.35 ppm, C_9_H_5_O_3_), respectively. The fragment ion at *m*/*z* 151.0393 (0.28 ppm, C_8_H_7_O_3_) was produced by [^1,4^A^−^ − C9H16]^−^, and the fragment ion at *m*/*z* 137.0235 (0.19 ppm, C_7_H_5_O_3_) was produced by [^1,4^A^−^ −·C9H15 −·CH3]^−^. These ions were useful for identifying metabolites.

### 2.5. Identification of the Prototype (Kuraridin, M0) and Metabolites (M1–M19) in Rat Plasma

**Figure 3:**
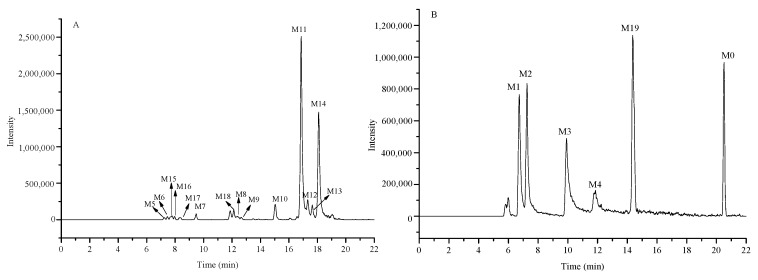
High-resolution EIC in 5 ppm for the metabolites in rat plasma (**A**) *m*/*z* 453.1919, 613.2291; (**B**) *m*/*z* 437.1970, 789.2612.

A total of 20 constituents were identified including the prototype and 19 metabolites by exact mass weights, ion spectra from MS^2^ data and reference standards. The chromatogram of them wasshown in Figure 3, and detailed MS^2^ spectra information was listed in Table 6.

#### 2.5.1. Identification of the Prototype (M0) in Rat Plasma

M0 was eluted at 20.51 min with the molecular ion of *m*/*z* 437.19815 (2.72 ppm, C_26_H_29_O_6_). It was unambiguously identified as kuraridin by comparing the retention time and fragment ions in the MS^2^ spectra with an authentic reference standard.

#### 2.5.2. Identification of Metabolites M1–M4

Metabolites M1–M4 were eluted at 6.74, 7.26, 9.91, and 11.87 min, respectively. The [M − H]^−^ ions of them showed at *m*/*z* 789.2626 (1.82 ppm, C_38_H_45_O_18_), *m*/*z* 789.2622 (1.36 ppm, C_38_H_45_O_18_), *m*/*z* 789.2623 (1.51 ppm, C_38_H_45_O_18_), *m*/*z* 789.2619 (0.97 ppm, C_38_H_45_O_18_), respectively. In their ESI-MS/MS spectra, the fragment ions [M − H − GluA]^−^ at *m*/*z* 613.22, and [M − H − 2GluA]^−^ at *m*/*z* 437.19 were found, indicating that these metabolites were diglucuronidation products of kuraridin.

#### 2.5.3. Metabolites M5–M14

Metabolites M5–M14 generated the same [M − H]^−^ ions at *m*/*z* 613.23 (C_32_H_37_O_12_). These metabolites produced the fragment ions at *m*/*z* 275.16, *m*/*z* 161.02, which were consistent with kuraridin. The fragment ion at *m*/*z* 437.19 was 176 Da less than that of the parent ion in the spectra, which showed the present of glucuronide in. Therefore, metabolites M5–M14 were identified as glucuronidation products of kuraridin.

#### 2.5.4. Metabolites M15–M18

Metabolites M15, M16, and M17 were eluted at 7.78, 7.94, and 8.40 min respectively. They generated the [M − H]^−^ ions at *m*/*z* 453.1926 (1.58 ppm, C_26_H_29_O_7_), *m*/*z* 453.1925 (1.38 ppm, C_26_H_29_O_7_), 453.1928 (1.93 ppm, C_26_H_29_O_7_), 16 Da more than that of kuraridin, suggesting the presence of a hydroxyl group. The fragment ion in the MS^2^ spectra of M15, M16, M17 was detected at *m*/*z* 291.16, which suggested that hydroxylation took place on the A-ring. The fragment ion was detected at *m*/*z* 151.03, which strongly suggested that hydroxylation took place on the prenyl moiety. M18 showed a [M − H]^−^ ion at *m*/*z* 453.1924 (1.24 ppm, C_26_H_29_O_7_): in the MS^2^ spectrum, the major daughter ions at *m*/*z* 275.1658 (^1,4^A^−^) and 177.0187 (^1,4^B^−^) were attributed to the 1,4-cleaveage of the C-ring. The ion at *m*/*z* 149.0237 was considered to correspond to a natural loss of CO from the ion at *m*/*z* 177.0187. The fragment ion at *m*/*z* 137.0235 was produced by the loss of ·C_9_H_15_ + ·CH_3_ from the ion at *m*/*z* 275.1658. Finally, M18 was identified as kushenol N by comparing the retention time and fragment ions in the MS^2^ spectra with an authentic reference standard [11].

#### 2.5.5. Metabolite M19

Metabolite M19 was eluted at 14.37 min with a quasi-molecular ion at *m*/*z* 437.19748 (1.19 ppm, C_26_H_29_O_6_). The fragment ions of M19 were the same as those of kuraridin, which was consistent with the literature [11]. First, the parent ion produced the main fragment ions at *m*/*z* 161.0236(^1,4^B^−^) and 275.1656(^1,4^A^−^). Then, the fragment ions at *m*/*z* 137.0235 and 151.0392 was yielded from the ion at *m*/*z* 275.1656. Finally, M19 was unambiguously identified as kurarinone by comparing the retention time and fragment ions in the MS^2^ spectra with authentic reference standard. The appearance of the 3 metabolite M19 suggested that kuraridin could be converted into kurarinone by cyclization in vivo.

### 2.6. Proposed Metabolic Pathways of Kuraridin

**Scheme 2:**
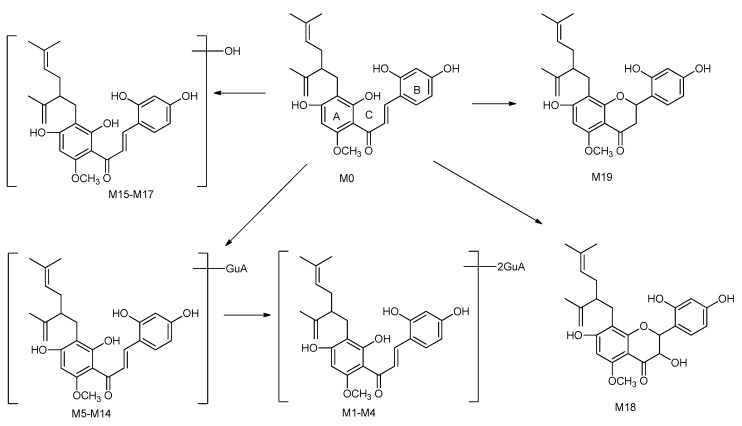
The proposed major metabolic pathways of kuraridin in rat plasma.

In this study, a total of 19 metabolites of kuraridin were identified or tentatively characterized from rat plasma. The proposed major metabolic pathways of kuraridin in rat plasma are shown in Scheme 2. In summary, hydroxylation and glucuronidation were the major drug clearance pathways of kuraridin in rat.

## 3. Materials and Methods

### 3.1. Chemicals and Reagents

**Figure 4:**
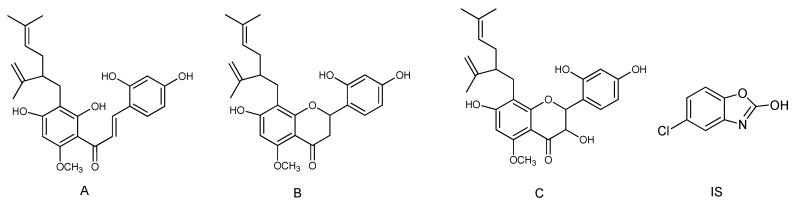
**Figure** **4.** Chemical structure of kuraridin (**A**); kurarinone (**B**); kushenol N (**C**) and chlorzoxazone (**IS**).

Kuraridin, kurarinone and kushenol N (Figure 4) was isolated and purified from the ethyl acetate extract of *Sophora flavescens* Ait [12]. Chlorzoxazone (Figure 4) used as an internal standard (IS) was obtained from the Guangzhou Institute for Drug Control (Guangzhou, China). The purity of the three flavonoid standards and IS were over 98.0%. LC-MS grade acetonitrile, ethyl acetate, methanol and water were supplied by Fisher Scientific (Hampton, VA, USA). Formic acid was procured from Kermel Chemical Reagents Co., Ltd. (Tianjin, China). All other chemicals and reagents were of analytical grade. Solid-phase extraction cartridges were Oasis HLB cartridges (1 cc/30 mg, 30 μm Waters, Milford, MA, USA).

### 3.2. LC-MS/MS Conditions

The LC-MS/MS system consisted of an Ultimate 3000 LC system (Dionex; Sunnyvale, CA, USA) and an Quadrupole-Exactive Orbitrap-Mass Spectrometry (Thermo Scientific, Bremen, Germany), which was equipped with an electro-spray ionization (ESI) source. Data acquisition were carried out by Xcalibur 4.0 workstation software. Chromatographic separation was achieved on a Hypersil GOLD UHPLC C18 column (1.9 µm, 2.1 × 100 mm, Thermo Scientific, Waltham, MA, USA) coupled to a guard cartridge (Hypersil GOLD C18, 2.1 × 5 mm, 1.9 µm, Thermo Scientific, Waltham, MA, USA) and the column temperature was maintained at 30 °C. The autosampler was conditioned at 4 °C.

The ESI source was operated in the negative ion mode, and the parameters were as follows: sheath gas flow rate of 15 L/min, auxiliary gas flow rate of 5 L/min, electrospray voltage, 3.0 kV; capillary temperature, 350 °C; S-lens RF level, 55; the collision energy, 15, 35, and 55%. In the full scan, the data were collected from *m*/*z* 100 to 1000 at 70,000 resolution with automatic gain control (AGC) target 1.0 × 10^6^. The quantification was performed using selected ion monitoring (SIM) mode with ions of [M − H]^−^ at *m*/*z* 423.19 for kuraridin and *m*/*z* 167.98 for IS. 

### 3.3. Pharmacokinetic Study

#### 3.3.1. Preparation of Calibration Standards and Quality Control (QC) Samples

Kuraridin and IS were dissolved separately in methanol to get a stock solution concentration of 100 μg/mL. Then the stock solution of kuraridin was diluted with methanol to prepare working solutions. The working solutions were further diluted with blank rat plasma to prepare calibration standards and QC samples. Final concentrations of the calibration standard were 1, 2, 10, 20, 100, 200 and 500 ng/mL for kuraridin in plasma. QC samples at the plasma lower limit of quantification (LLOQ), low, medium and high concentrations were as follow: 1 (LLOQ), 2 (low), 20 (medium) and 400 (high) ng/mL. The IS working solution (300 ng/mL) was prepared by diluting the stock solution with methanol. 

#### 3.3.2. Sample Preparation

After thawing at room temperature, 100 μL of plasma sample were transferred to 1.5 mL centrifuge tube. 20 μL of IS solution (300 ng/mL) and 20 μL of methanol were mixed followed by the addition of 1.0 mL ethyl acetate-acetonitrile (1:3), and then the tubes were vortex mixed for 2.0 min. After centrifugation at 13,000 rpm for 10 min at 4 °C, the supernatant was evaporated to dryness at 40 °C. The residue was reconstituted in 100 μL of methanol and vortexed for 1 min to make sure the residue was well dissolved. After centrifugation at 14,000 rpm for 15 min at 4 °C, 2 μL of the supernatant was subjected to LC-MS/MS for the analysis. The mobile phase was composed of 0.1% formic acid in water (solvent A) and acetonitrile (solvent B) at a flow rate of 0.3 mL/min. The gradient was set as follows: 0–2 min, 35–61% B; 2–5 min, 61–62% B; 5–7 min, 62–80% B; 7–8 min, 80–35% B.

#### 3.3.3. Method Validation

The proposed LC-MS/MS method was fully validated in this study included specificity, linearity, precision, accuracy, recovery, matrix effect and stability according the FDA bioanalytical method validation guide.

##### Specificity

The specificity was determined by comparing the chromatograms of blank plasma samples, blank plasma samples spiked with the kuraridin and IS and plasma samples obtained after an oral dose.

##### Linearity and Lower Limits of Quantification (LLOQ)

The calibration curve was established by plotting the peak areas ratios (kuraridin/IS) (Y) and plasma concentrations (X). The 1/x^2^ was chosen as the weighting factor used for determining linear regression. The LLOQ was defined as the lowest concentration in the calibration curve with an acceptable accuracy and precision.

##### Precision and Accuracy

The precision and accuracy were carried out through analyzing six replicates of QC samples at four concentration levels (1, 2, 20 and 400 ng/mL) on the same day (intra-day) and on the three consecutive days (inter-day). The Relative standard deviation (RSD) and relative error (RE) was used to evaluate precision and accuracy, respectively.

##### Extraction Recovery and Matrix Effect

The extraction recovery and matrix effect of kuraridin were tested by analyzing blank plasma spiked with kuraridin at three concentration levels (2, 20 and 400 ng/mL) with six replicates. Extraction recovery was calculated by comparing the peak area of blank plasma spiked with kuraridin before extraction with those of the extracted blank plasma spiked with kuraridin. The matrix effect was evaluated by comparing the analyte/internal standard peak ratios dissolved with blank matrix extract against those dissolved with the mobile phase at high, medium and low levels. The extraction recovery and matrix effect of the IS at 300 ng/mL were evaluated using the same method. 

##### Stability

The stability of kuraridin was assessed by analyzing the QC samples at three concentration levels (*n* = 6) under the follow conditions: 21 days at −80 °C for long-term stability, 4 h at room temperature for short-term stability, three freeze at −20 °C and thaw cycles for freeze-thaw stability, and 24 h storage in the autosampler (4 °C) for post-preparative stability.

#### 3.3.4. Application in Pharmacokinetic Study

Male Sprague-Dawley rats (300 ± 20 g) were supplied by the Laboratory Animal Center of the Traditional Chinese Medicine University of Guangzhou (Guangzhou, China) and were housed in a temperature-controlled environment with temperature at 25 ± 2 °C, humidity at 70 ± 5% for a week. Animal study was undertaken with the Guidelines of the Experimental Animal Center of Guangdong Pharmaceutical University, and was approved by the Animal Ethics Committee of the institution (Ethic Approval No.: GDPU 2016067). Before experiment, six rats were deprived of food overnight (12 h) but allowed free access to water. All rats were orally administrated a dose of kuraridin suspension (5 mg/mL in 0.5% carboxymethylcellulose sodium aqueous solution) at 10.2 mg/kg. Blood samples (about 0.4 mL) were collected in heparinized centrifuge tubes through retro orbital sinus at 0, 0.083, 0.25, 0.5, 0.75, 1, 2, 4, 6, 8, 10, 12 and 24 h after administration. Blood samples were centrifuged (4000 rpm for 10 min at 4 °C) immediately to obtain plasma sample. The plasma samples were stored at −80 °C until analysis. All animals were free access to water throughout the experiment and free access to food after 6 hours after administration. All animals are not fixed throughout the experiment.

### 3.4. Metabolites Study

#### 3.4.1. Animals and Drug Administration

Male Sprague-Dawley rats were randomly divided into two groups: control group (*n* = 3) and drug group (*n* = 3), control group for blank plasma. All animals were fasted for 12 h free access to water before the experiment. Animal study was undertaken with the Guideline of Experimental Animal Center of Guangdong Pharmaceutical University, and was approved by the Animal Ethics Committee of the institution (Ethic Approval No.: GDPU 2016067). The drug group was orally administered a single dose of kuraridin suspension (5 mg/mL in 0.5% carboxymethylcellulose sodium aqueous solution) at 50 mg/kg, and the control group were orally administered the equivalent 0.5% carboxymethylcellulose sodium aqueous solution. The blood samples were collected from retro orbital sinus in heparinized centrifuge tube at 0.5, 1, 2 and 4 h after the oral administration, respectively [13]. 

#### 3.4.2. Sample Extraction

The plasma sample was pretreated by a solid-phase extraction method (Waters Oasis HLB cartridge, 1 cc/30 mg, 30 μm). All plasma samples at each time point from the same group of rats were combined into one sample so as to eliminate the individual variability. Before extraction, the solid phase column was activated with 5 mL of methanol and 5 mL of purified water successively. The 0.3 mL plasma sample was loaded on a pre-activated Oasis HLB solid phase extraction C18 column, and the cartridge was washed with 5 mL water and 5 mL methanol successively. The methanol eluate was collected and evaporated to dry under N_2_. The residue was redissolved in 100 µL of methanol and centrifuged at 14,000 rpm for 15 min at 4 °C. A volume of 3 µL of supernatant was injected into the LC-MS/MS for analysis. The mobile phase was composed of water (solvent A) and methanol (solvent B) at a flow rate of 0.2 mL/min with a linear gradient as follows: 0–3 min, 10–60% B; 3–10 min, 60–65% B; 10–25 min, 65–85% B; 25–27 min, 85–90% B; 27–30 min, 90% B.

#### 3.4.3. Peak Selections and Data Processing

A Thermo Xcalibur 4.0 workstation was used for the data acquiring and processing. In order to get more fragment ions of the metabolites, the peaks detected with intensity over 10,000 were selected for identification. The chemical formulas for all parent ions of the selected peaks were calculated from the accurate mass using a formula predictor by setting the parameters as follows: C [0–40], H [0–50], O [0–50], S [0–4], N [0–4], Cl [0–4]. 

## 4. Conclusions

Although prenylated flavonoids from *S. flavescens* have a variety of biological activities, little is known about the metabolism of these compounds. Therefore, the current study was designed to use an ultra-performance liquid chromatography/tandem mass spectrometry (UHPLC-MS/MS) method for the quantification and characterization metabolites in rat plasma after oral administration of kuraridin. 

In the present study, a total of 20 components, including the prototype and 19 metabolites have been identified or tentatively characterized from rat plasma, of which three kurarinone, kushenol N, kuraridin were unambiguously identified by comparing their retention times and mass spectra with those of reference standards, while the other 17 compounds were tentatively identified on the basis of their MS^2^ fragmentation behaviors and exact mass information from literature. It is concluded the developed UHPLC-QExactive MS method with high sensitivity and resolution is suitable for identifying and characterizing the metabolites of kuraridin. To the best of our knowledge, it is the first time that kuraridin could be converted into kurarinone and kushenol N in vivo, which indicated that interconversions of the drug occurred after the oral administration, and the result may be a good explanation why so many metabolites (M5–M14) generated the same [M − H]^−^ ions at *m*/*z* 613.23. Due to the limitations of the quantity of standard compounds, this study failed to quantify the metabolites (kurarinone and kushenol N) at the same time, in the future study, we should carry out an in depth study of the pharmacokinetics of the metabolites of kuraridin.

The proposed metabolic pathways of kuraridin in rat are shown in Scheme 2. The results revealed that glucuronidation and hydroxylation were the main metabolic pathways of kuraridin and the results will provide essential data for the pharmacokinetics and clinical application of *S. flavescens.*

Moreover, in the study, a rapid and sensitive UHPLC-MS/MS method for determination of kuraridin was established and validated, which was successfully applied to the pharmacokinetic studies of kuraridin after oral administration. Excellent linearity, sensitivity, precision and accuracy were achieved. This pharmacokinetic study could be useful for future PK-PD study and the results would provide a reference for the clinical development and clinical research of kuraridin.

## Figures and Tables

**Table 1:** Calibration curves for kuraridin in rat plasma.

Compound	Linear Range (ng/mL)	Regression Equation	r^2^	LLOQ
Kuraridin	1–500	Y = 7.063 × 10^−3^ X− 2.585 × 10^−3^	0.9931	1

**Table 2:** Precision and accuracy data of kuraridin in rat plasma (*n* = 6).

Component	Concentration	Precision RSD (%)		Accuracy RE (%)	
	(ng/mL)	Intra-Day	Inter-Day	Intra-Day	Inter-Day
Kuraridin	1	8.95	2.18	4.12	2.09
	2	5.21	6.08	3.07	6.48
	20	4.03	1.38	−0.57	1.22
	400	4.35	3.50	−4.97	−6.27

**Table 3:** Recovery and matrix effect (%) data for the analytes in rat plasma (*n* = 6).

Components	Concentration (ng/mL)	Recovery (Mean ± S.D)	Matrix Effect (Mean ± S.D)
	2	90.1 ± 4.7	87.1 ± 4.5
Kuraridin	20	101.7 ± 5.7	87.4 ± 3.1
	400	100.4 ± 2.9	84.8 ± 4.6
IS	300	91.2 ± 2.9	90.1 ± 3.2

**Table 4:** Stability of kuraridin in rat plasma (*n* = 6).

Storage Conditions		High Concentrations	Medium Concentrations	Low Concentrations
Autosampler 4 °C for 12 h	RE (%)	1.27	7.66	−8.86
	RSD (%)	12.80	7.05	8.23
Short-term (at room temperature for 4 h)	RE (%)	3.29	10.01	14.31
	RSD (%)	12.23	5.51	10.68
Three freeze-thraw cycles	RE (%)	−9.97	8.1	10.82
	RSD (%)	10.20	4.31	7.07
Storage at −75 °C for 21 days	RE (%)	4.68	0.89	8.97
	RSD (%)	8.28	4.18	8.17

**Table 5:** Main pharmacokinetic parameters of kuraridin after oral administration (*n* = 6).

Parameters	Kuraridin
C_max_ (μg/L)	65.43 ± 6.54
T_max_ (h)	0.87 ± 0.14
T_1/2z_ (h)	5.54 ± 3.17
AUC_0–t_ (μgh/L)	284.05 ± 24.19
AUC_0–∞_ (μgh/L)	289.79 ± 24.08
MRT_0–t_ (h)	7.08 ± 0.86
MRT_0–∞_ (h)	8.79 ± 1.22

**Table 6:** Summary of kuraridin metabolites in rat plasma.

Peak	t_R_	Experimental	Theoretical	Error	Formula [M − H]^−^	MS/MS Fragment	Identification/Reactions
		Mass (*m*/*z*)	Mass (*m*/*z*)	(ppm)			
M1	6.74	789.2626	789.2612	1.82	C_38_H_45_O_18_	MS^2^[789]: 161.0235(100),613.2296(36),275.1656(33),437.1964(4)	Diglucuronidation
M2	7.26	789.2622	789.2612	1.36	C_38_H_45_O_18_	MS^2^[789]: 161.0235(100),613.2289(31),275.1659(31),437.1954(4)	Diglucuronidation
M3	9.91	789.2623	789.2612	1.51	C_38_H_45_O_18_	MS^2^[789]: 161.0235(100),275.1655(31),613.2299(28),437.1989(5)	Diglucuronidation
M4	11.87	789.2619	789.2612	0.97	C_38_H_45_O_18_	MS^2^[789]: 161.0235(100),789.2916(36),275.1658 (28),613.2286(16)	Diglucuronidation
M5	7.44	613.2299	613.2291	1.32	C_32_H_37_O_12_	MS^2^[613]: 161.0237(100),275.1653(41),437.1976(5)	Glucuronidation
M6	7.66	613.2302	613.2291	1.91	C_32_H_37_O_12_	MS^2^[613]: 161.0236(100),275.1659(28),137.0235(23),437.1969(5)	Glucuronidation
M7	9.48	613.2302	613.2291	1.91	C_32_H3_7_O_12_	MS^2^[613]: 161.0237(100),275.1659(26),151.0391(17)	Glucuronidation
M8	12.40	613.2299	613.2291	1.32	C_32_H_37_O_12_	MS^2^[613]: 161.0236(100),275.1653(13),151.0394(13)	Glucuronidation
M9	12.63	613.2296	613.2291	0.82	C_32_H_37_O_12_	MS^2^[613]: 161.0237(100),275.1658(21),137.0236(6)	Glucuronidation
M10	15.02	613.2297	613.2291	1.23	C_32_H_37_O_12_	MS^2^[613]: 161.0236(100),275.1657(39),437.1979(15)	Glucuronidation
M11	16.85	613.2300	613.2291	1.61	C_32_H_37_O_12_	MS^2^[613]: 161.0236(100),275.1658(23),437.1980(7)	Glucuronidation
M12	17.31	613.2301	613.2291	1.71	C_32_H_37_O_12_	MS^2^[613]: 161.0237(100),275.1658(18),437.1980(14)	Glucuronidation
M13	17.64	613.2302	613.2291	1.91	C_32_H_37_O_12_	MS^2^[613]:161.0237(100),275.1662(19),437.1980(11)	Glucuronidation
M14	18.08	613.2301	613.2291	1.71	C_32_H_37_O_12_	MS^2^[613]: 161.0236(100),275.1658(26),151.0393(13),437.1979(5)	Glucuronidation
M15	7.78	453.1926	453.1919	1.58	C_26_H_29_O_7_	MS^2^[453]: 161.0237(100),151.0392(36),291.1607(16)	Hydroxylation
M16	7.94	453.1925	453.1919	1.38	C_26_H_29_O_7_	MS^2^[453]: 161.0236(100),151.0392(20),291.1609(15),137.0235(13)	Hydroxylation
M17	8.40	453.1928	453.1919	1.93	C_26_H_29_O_7_	MS^2^[453]: 161.0236(100),137.0236(36),291.1610(25),151.0393(13)	Hydroxylation
M18	12.11	453.1924	453.1919	1.24	C_26_H_29_O_7_	MS^2^[453]: 149.0237(100),177.0187(96),275.1658(63),137.0234(49),151.0394(38)	Kushenol N
M19	14.37	437.1975	437.19696	1.19	C_26_H_29_O_6_	MS^2^[437]: 161.0236(100),275.1656(33),137.0235(24),151.0392(14)	Kurarinone
M0	20.51	437.1982	437.19696	2.72	C_26_H_29_O_6_	MS^2^[437]: 161.0237(100),275.1657(39),137.0235(31),151.0393(20)	Kuraridin

## References

[1] He X., Fang J., Huang L., Wang J., Huang X. (2015). *Sophora flavescens* Ait., Traditional usage, phytochemistry and pharmacology of an important traditional Chinese medicine. J. Ethnopharmacol..

[2] Liu B., Shi R.B., Chen S. (2006). HPLC determination of xanthohumol and kuraridin in *Sophora flavescens* Ait. and the effective fraction of Kushen decoction. Chin. J. Pharm. Anal..

[3] Sun M., Han J., Duan J., Cui Y., Wang T., Zhang W., Liu W., Hong J., Yao M., Xiong S. (2007). Novel antitumor activities of Kushen flavonoids in vitro and in vivo. Phytother. Res..

[4] Ryu S.Y., Choi S.U., Kim S.H. (1997). In vitro antitumor activity of flavoniod from *Sophora flavascens*. Phytother. Res..

[5] Lee H.S., Ko H.R., Ryu S.Y., Oh W.K., Kim B.Y., Ahn S.C., Mheen T.I., Ahn J.S. (1997). Inhibition of phospholipase C_γ_1 by the prenylated flavonoids from *Sophora flavescens*. Planta Medica.

[6] Kim J.H., Ryu Y.B., Kang N.S., Lee B.W., Heo J.S., Jeong I.Y., Park K.H. (2006). Glycosidase inhibitory flavonoids from *Sophora flavescens*. Biol. Pharm. Bull..

[7] Sato S., Takeo J., Aoyama C., Kawahara H. (2007). Na^+^-glucose cotransporter (SGLT) inhibitory flavonoids from the roots of *Sophora flavescens*. Bioorg. Med. Chem..

[8] Sasaki T., Li W., Higai K., Quang T.H., Kim Y.H., Koike K. (2014). Protein Tyrosine Phosphatase 1B Inhibitory Activity of Lavandulyl Flavonoids from Roots of *Sophora flavescens*. Planta Medica.

[9] Chan B.C., Yu H., Wong C.W., Lui S.L., Jolivalt C., Ganem-Elbaz C., Paris J.M., Morleo B., Litaudon M., Lau C.B. (2012). Quick identification of kuraridin, a noncytotoxic anti-MRSA (methicillin-resistant *Staphylococcus aureus*) agent from *Sophora flavescens* using high-speed counter-current chromatography. J. Chromatogr. B.

[10] Kwon H.J., Jeong J.H., Lee S.W., Ryu Y.B., Jeong H.J., Jung K., Lim J.S., Cho K.O., Lee W.S., Rho M.C. (2015). In vitro anti-reovirus activity of kuraridin isolated from *Sophora flavescens* against viral replication and hemagglutination. J. Pharmacol. Sci..

[11] Zhang Y., Zhang P., Cheng Y. (2008). Structural characterization of isoprenylated flavonoids from Kushen by electrospray ionization multistage tandem mass spectrometry. J. Mass Spectrom..

[12] Huang R., Liu Y., Zhao L.L., Chen X.X., Wang F., Cai W., Chen L. (2017). A new flavonoid from *Sophora flavescens* Ait. Nat. Prod. Res..

[13] Havsteen B.H. (2002). The biochemistry and medical significance of the flavonoids. Pharmacol. Ther..

